# Clinical Impact of Preoperative Tonsil and Adenoid Size on Symptomatic Outcomes Following Adenotonsillectomy in Pediatric Patients

**DOI:** 10.7759/cureus.47093

**Published:** 2023-10-16

**Authors:** Yahya A Fageeh

**Affiliations:** 1 Otolaryngology - Head and Neck Surgery, College of Medicine, Taif University, Taif, SAU

**Keywords:** noisy breathing, mouth breathing, snoring, persistent symptoms, grading scale, obstructive sleep apnea, upper airway obstruction, tonsillectomy, adenoidectomy, adenotonsillar hypertrophy

## Abstract

Background

Adenotonsillar hypertrophy is a common clinical problem in pediatric patients. Adenotonsillectomy is a surgical intervention to remove airway obstruction and alleviate symptoms. However, some children continue to experience persistent symptoms after surgery.

Objective

This study aimed to investigate the relationship between preoperative tonsils and adenoid size and the persistence of symptoms, including snoring, mouth breathing, noisy breathing, and sleep apnea, after adenotonsillectomy in pediatric patients.

Method

This study was conducted in Taif, Saudi Arabia, and included 109 pediatric patients aged three to 14 years who underwent adenotonsillectomy. Data on preoperative and postoperative symptoms were collected through patient records and follow-up surveys. Tonsil and adenoid size were assessed using the Brodsky scale and endoscopic grading scales, respectively. Statistical analysis was performed using SPSS Version 26 (IBM Corp., Armonk, NY).

Results

The most prevalent presenting symptoms were snoring, mouth breathing, and noisy breathing. Tonsil size grades 3+ and 4+ were significantly more prevalent than the other grades (p<0.05). Adenoid size grades 3 and 4 were also significantly more prevalent than the other grades (p<0.05). Significant associations were observed between tonsil and adenoid size grades and specific presenting symptoms, such as snoring, mouth breathing, and noisy breathing. No significant correlations were found between preoperative tonsil or adenoid size and postoperative persistent symptoms.

Conclusion

While tonsil and adenoid size are essential factors in determining the need for surgery, they may not predict postoperative resolution of symptoms. A comprehensive evaluation of various clinical factors is necessary to understand the persistence of symptoms after surgery. Although adenotonsillectomy is an effective treatment for upper airway obstruction in pediatric patients, some individuals may experience residual symptoms.

## Introduction

Adenotonsillar hypertrophy is a common clinical problem in children worldwide, including Saudi Arabia [1]. These vital lymphoid tissues undergo significant growth in the first years of life, peaking around the age of five years in response to increasing immune activity [2]. Children exposed to chronic infections, reflux, and passive cigarette smoke can develop adenotonsillar hypertrophy, which obstruct the airway due to limited space in the nasopharynx and oropharynx [3]. Children with adenotonsillar hypertrophy commonly snore during sleep but may not experience breathing difficulties while awake. However, they may display symptoms such as speaking with a nasal tone, breathing through their mouth, and having nasal discharge. However, these symptoms are not determining factors for surgery [2]. When a child sleeps, the pharyngeal muscles relax, which can cause enlarged tonsils and adenoids to block the airway. This can lead to temporary pauses in airflow, called apneas or hypopneas, and may result in decreased oxygen saturation of blood, leading to arousal from sleep. The recurrent partial or complete obstruction of the airway during sleep is called obstructive sleep apnea (OSA). If left untreated, OSA can cause serious health problems such as pulmonary hypertension, respiratory acidosis, and right heart failure [3,4]. Adenotonsillectomy can remove airway obstruction and prevent complications and associated preoperative symptoms [5].

When evaluating the size of tonsils, the most commonly used scale is the 0-4 scale developed by Brodsky et al. This is a reliable method for measuring the severity of tonsillar enlargement [6]. Additionally, the size of adenoids can be assessed using an endoscopic grading scale as per the study by Cassano et al. [7]. Numerous studies have demonstrated a clear correlation between the preoperative size of tonsils and adenoids and obstructive symptoms [7-10].

It has been observed that the majority of children experience relief from upper airway obstruction following adenotonsillectomy [11,12]. However, there are instances where obstructive symptoms persist, and the underlying causes of this phenomenon still need to be fully investigated [13,14].

A comprehensive review of the existing literature has revealed a significant gap, with no prior investigations examining the association between preoperative tonsils and adenoid size and the subsequent persistence of obstructive symptoms after surgery. Consequently, limited data are available concerning the relationship between tonsils and adenoid size and symptomatic improvement following surgery in children, whether within the national context of Saudi Arabia or globally. The present study seeks to bridge this knowledge gap by exploring the connection between preoperative tonsils and adenoid size and the persistence of associated symptoms following surgical intervention.

## Materials and methods

This observational cohort study was conducted in Taif, Saudi Arabia, and focused on pediatric patients aged three to 14 years who underwent adenotonsillectomy from February 2019 to February 2020. The data were collected preoperatively and two years after surgery.

Study participants

The criteria for selection involved observing children who displayed obstructive symptoms, such as snoring, mouth breathing, noisy breathing, and sleep apnea. Only children whose parents gave informed consent were allowed to participate. Those who did not exhibit obstructive symptoms or whose parents did not agree to participate were excluded. The study excluded children with syndromes such as Down syndrome, craniofacial disorders, congenital anomalies, respiratory diseases, or neuromuscular disorders. In total, 109 children met these strict criteria and were enrolled in the study. All participants had their tonsils and adenoids removed simultaneously using traditional extracapsular tonsillectomy and curettage technique for adenoidectomy.

Data collection

A comprehensive review of patient records was conducted to acquire data on preoperative symptoms. This survey encompassed information on the child's age at the time of surgery, gender, and a range of symptoms, including snoring, mouth breathing, noisy breathing, sleep apnea, hearing loss, and sore throat frequency. Postoperative symptom data were collected through a follow-up phone survey with participants' parents two years following surgery.

Tonsil and adenoid size assessment

Tonsils and adenoid size were assessed using established grading scales. The tonsils size was evaluated based on Brodsky grading scale, which categorizes obstruction into four grades: "grade 1+" (0% to 25% airway obstruction), "grade 2+" (25% to 50% obstruction), "grade 3+" (50% to 75% obstruction), and "grade 4+" (75% to 100% obstruction) [6].

Adenoid size was evaluated using an endoscopic grading scale as per study by Cassano et al. that delineated adenoid enlargement into four distinct categories: grade 1, representing adenoids occupying less than 25% of the choanal area; grade 2, where adenoids occupied 25% to 50% of the choanal area; grade 3, indicating adenoids occupying 50% to 75% of the choanal area; and grade 4, denoting adenoids occupying 75% to 100% of the choanal area [7,15].

Statistical analysis

Data were analyzed using the Statistical Package for the Social Sciences (SPSS) Version 26 (IBM Corp., Armonk, NY). Descriptive data were presented as numbers and frequencies. The chi-squared test (χ2) test was used to assess relationships between variables. Quantitative data were expressed as mean ± SD. The significance level was set at a p-value of less than 0.05.

Ethical considerations

The research was approved by the Ethical Research Committee of Taif University, Saudi Arabia (approval number 940-36-0130). Participants were informed of the study objectives, gave their consent, and were assured of confidentiality during data collection.

## Results

**Figure 1:**
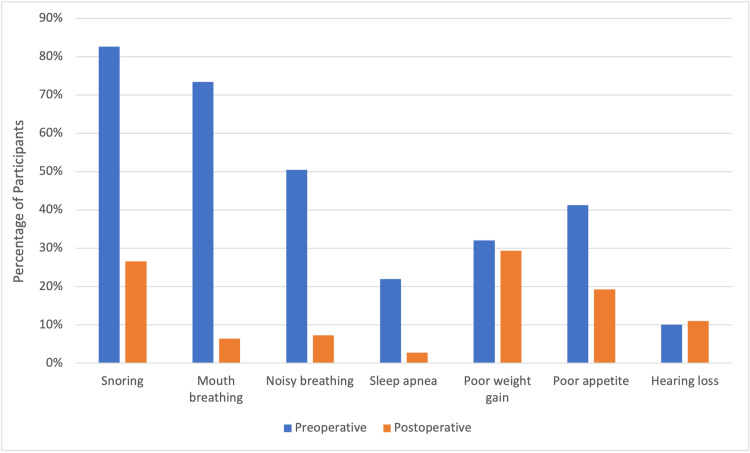
Distribution of symptoms before and after adenotonsillectomy (n=109)

The study included 109 children with a mean age of 6.93 years (±2.57), with 57 (52.3%) being males. Snoring was the most prevalent presenting symptom in 90 (82.6%) children. After surgery, its prevalence decreased significantly to 29 (26.6%). Similarly, the prevalence of mouth breathing decreased from 80 (73.4%) to 7 (6.4%) after surgery. Daytime noisy breathing and sleep apnea also showed significant reductions, from 55 (50.5%) to 8 (7.3%) and 24 (22%) to 3 (2.8%), respectively. The prevalence of poor appetite decreased from 45 (41.3%) to 21 (19.3%) after surgery. Although other symptoms, such as poor weight gain and hearing loss, persisted after surgery, their prevalence did not change significantly (Figure 1). Among the participants, 32 (29.4%) experienced sore throat attacks fewer than five times per year, whereas 77 (70.6%) suffered from these attacks more frequently, with a mean symptom duration of 28.25 (±11.72) months.

Tonsil size grades among the children were distributed as follows: five (4.6%) had grade 1+, 26 (23.9%) had grade 2+, 48 (44%) had grade 3+, and 30 (27.5%) had grade 4+. Regarding adenoid size, 23 (21.1%) were grade 1, 25 (22.9%) were grade 2, 37 (33.9%) were grade 3, and 24 (22%) were grade 4.

Tonsil size grades 3+ and 4+ were significantly more prevalent than the other grades (p<0.05). Moreover, significant associations were observed between tonsil size grades and specific presenting symptoms, including snoring, mouth breathing, noisy breathing, poor weight gain, poor appetite, and hearing loss. However, no significant relationships were found between tonsils grades and sleep apnea or frequent sore throat (Table 1). No significant correlations were found between preoperative tonsil size and all postoperative persistent symptoms (Table 2).

**Table 1:** Relationship between preoperative tonsil grades and symptoms before adenotonsillectomy No., number; %, percent, χ2, chi-squared test

Parameter	Tonsil grade 1+	Tonsil grade 2+	Tonsil grade 3+	Tonsil grade 4+	χ2	P-value
	No. (%)	No. (%)	No. (%)	No. (%)		
Snoring						
Yes	2 (2.2)	20 (22.2)	39 (43.3)	29 (32.2)	11.07	0.011
No	3 (15.8)	6 (31.6)	9 (47.4)	1 (5.3)		
Mouth breath						
Yes	2 (2.5)	15 (18.8)	37 (46.3)	26 (32.5)	10.9	0.012
No	3 (10.3)	11 (37.9)	11 (37.9)	4 (13.8)		
Noisy breath						
Yes	1 (1.80	13 (23.6)	18 (32.7)	23 (41.8)	13.32	0.004
No	4 (7.4)	13 (24.1)	30 (55.6)	7 (13)		
Sleep apnea						
Yes	0 (0.0)	5 (20.8)	9 (37.5)	10 (41.7)	4.06	0.25
No	5 (5.9)	21 (24.7)	39 (45.9)	20 (23.5)		
Poor weight gain						
Yes	1 (2.9)	5 (14.3)	12 (34.3)	17 (48.6)	11.72	0.008
No	4 (5.4)	21 (28.4)	36 (48.6)	13 (17.6)		
Poor appetite						
Yes	1 (2.2)	7 (15.6)	18 (40)	19 (42.2)	9.44	0.024
No	4 (6.3)	19 (29.7)	30 (46.9)	11 (17.2)		
Hearing loss						
Yes	0 (0.0)	1 (9.1)	3 (27.3)	7 (63.6)	8.25	0.041
No	5 (5.1)	25 (25.5)	45 (45.9)	23 (23.5)		
Sore throat frequency/year						
˂5 times	0 (0.0)	8 (25)	14 (43.8)	10 (31.3)	2.33	0.5
≥5 times	5 (6.5)	18 (23.4)	34 (44.2)	20 (26)		

**Table 2:** Relationship between preoperative tonsil grades and symptoms two years after adenotonsillectomy No., number; %, percent; χ2, chi-squared test

Parameter	Tonsil grade 1+	Tonsil grade 2+	Tonsil grade 3+	Tonsil grade 4+	χ2	P-value
	No. (%)	No. (%)	No. (%)	No. (%)		
Snoring						
Yes	1 (3.4)	10 (34.5)	7 (24.1)	11 (37.9)	7.09	0.06
No	4 (5)	16 (20)	41 (51.3)	19 (23.8)		
Mouth breath						
Yes	1 (14.3)	2 (28.6)	3 (42.9)	1 (14.3)	2.08	0.55
No	4 (3.9)	24 (23.5)	45 (44.1)	29 (28.4)		
Noisy breath						
Yes	0 (0.0)	1 (12.5)	4 (50)	3 (37.5)	1.24	0.74
No	5 (5)	25 (24.8)	44 (43.6)	27 (26.7)		
Sleep apnea						
Yes	1 (33.3)	0 (0.0)	2 (66.7)	0 (0.0)	7.5	0.05
No	4 (3.8)	26 (24.5)	46 (43.4)	30 (28.3)		
Poor weight gain						
Yes	0 (0.0)	12 (37.5)	14 (43.8)	6 (18.8)	6.88	0.07
No	5 (6.5)	14 (18.2)	34 (44.2)	24 (31.2)		
Poor appetite						
Yes	2 (9.5)	8 (38.1)	6 (28.6)	5 (23.8)	5.13	0.16
No	3 (3.4)	18 (20.5)	42 (47.7)	25 (28.4)		
Hearing loss						
Yes	0 (0.0)	3 (25)	6 (50)	3 (25)	0.76	0.85
No	5 (5.2)	23 (23.7)	42 (43.3)	27 (27.8)		
Sore throat frequency/year						
˂5 times	0 (0.0)	2 (12.5)	8 (50)	6 (37.5)	2.7	0.44
≥ 5 times	5 (5.6)	24 (25.8)	40 (43)	24 (25.8)		

In the context of adenoid size, grades 3 and 4 exhibited significantly higher prevalence compared to other grades (p<0.05). A significant correlation was also established between adenoid size and airway obstruction symptoms, including snoring, mouth breathing, noisy breathing, sleep apnea, poor weight gain, and poor appetite (Table 3). No significant correlations were found between preoperative adenoid size and all postoperative persistent symptoms (Table 4).

**Table 3:** Relationship between preoperative adenoid grades and symptoms before adenotonsillectomy No., number; %, percent; χ2, chi-squared test

Parameter	Adenoid grade 1	Adenoid grade 2	Adenoid grade 3	Adenoid grade 4	χ2	P-value
	No. (%)	No. (%)	No. (%)	No. (%)		
Snoring						
Yes	7 (7.8)	24 (26.7)	35 (38.9)	24 (26.7)	55.35	0.0001
No	16 (84.2)	1 (5.3)	2(10.5)	0 (0.0)		
Mouth breath						
Yes	4 (5)	22 (27.5)	35 (43.8)	19 (23.8)	48.59	0.0001
No	19 (65.5)	3 (10.3)	2 (6.9)	5 (17.2)		
Noisy breath						
Yes	4 (7.3)	14 (25.5)	20 (36.4)	17 (30.9)	14.54	0.002
No	19 (35.2)	11 (20.4)	17 (31.5)	7 (13)		
Sleep apnea						
Yes	0 (0.0)	3 (12.5)	6 (25)	15 (62.5)	31.58	0.0001
No	23 (27.1)	22 (25.9)	31 (36.5)	9 (10.6)		
Poor weight gain						
Yes	1 (2.9)	8 (22.9)	11 (31.4)	15 (42.9)	18.39	0.0001
No	22 (29.7)	17 (23)	26 (35.1)	9 (12.2)		
Poor appetite						
Yes	4 (8.9)	10 (22.2)	16 (35.6)	15 (33.3)	9.94	0.019
No	19 (29.7)	15 (23.4)	21 (32.8)	9 (14.1)		
Hearing loss						
Yes	0 (0.0)	3 (27.3)	3 (27.3)	5 (45.5)	5.89	0.117
No	23 (23.5)	22 (22.4)	34 (34.7)	19 (19.4)		

**Table 4:** Relationship between preoperative adenoid grades and symptoms two years after adenotonsillectomy No., number; %, percent; χ2, chi-squared test

Parameter	Adenoid grade 1	Adenoid grade 2	Adenoid grade 3	Adenoid grade 4	χ2	p-value
	No. (%)	No. (%)	No. (%)	No. (%)		
Snoring						
Yes	5 (17.2)	4 (13.8)	12 (41.4)	8 (27.6)	2.91	0.4
No	18 (22.5)	21 (26.3)	25 (31.3)	16 (20)		
Mouth breath						
Yes	4 (57.1)	1 (14.3)	2 (28.6)	0 (0.0)	6.56	0.08
No	19 (18.6)	24 (23.5)	35 (34.3)	24 (23.5)		
Noisy breath						
Yes	1 (12.5)	2 (25)	3 (37.5)	2 (25)	0.38	0.94
No	22 (21.8)	23 (22.8)	34 (33.7)	22 (21.8)		
Sleep apnea						
Yes	1 (33.3)	0 (0.0)	2 (66.7)	0 (0.0)	2.57	0.46
No	22 (20.8)	25 (23.6)	35 (33)	24 (22.6)		
Poor weight gain						
Yes	7 (21.9)	7 (21.9)	11 (34.4)	7 (21.9)	0.03	0.99
No	16 (20.8)	18 (23.4)	26 (33.8)	17 (22.1)		
Poor appetite						
Yes	3 (14.3)	5 (23.8)	6 (28.6)	7 (33.3)	2.31	0.51
No	20 (22.7)	20 (22.7)	31 (35.2)	17 (19.3)		
Hearing loss						
Yes	1 (8.3)	4 (33.3)	4 (33.3)	3 (25)	1.73	0.63
No	22 (22.7)	21 (21.6)	21 (21.6)	21 (21.6)		

## Discussion

This study explores the relationship between preoperative adenotonsillar hypertrophy grades and the persistence of symptoms after adenotonsillectomy in pediatric patients. The study's findings suggest that the preoperative size of tonsils and adenoids alone may not be a reliable predictor of postoperative symptom persistence. The study found no significant correlation between postoperative symptoms and the size of tonsils and adenoids before surgery. This outcome is consistent with previous studies that have examined the correlation between preoperative tonsils and adenoid grades and specific postoperative complications and effects [16,17].

This study indicates that snoring and mouth breathing are the most prevalent symptoms experienced by the pediatric patients we studied. These findings align with previous studies and highlight the impact of enlarged tonsils and adenoids on the pharyngeal airway, which can cause these symptoms [18]. Additionally, this study supports the connection between the preoperative size of adenoids and tonsils and the prevalence of these symptoms at the time of diagnosis [6,7].

Studies have shown that patients with larger adenoids and tonsils tend to experience more significant improvements in symptoms after undergoing adenotonsillectomy [15,19]. Removing airway obstruction through adenotonsillectomy consistently resolves associated preoperative symptoms [20-22]. It is not surprising that adenotonsillectomy remains the first-line treatment for pediatric upper airway problems, as it brings marked improvements in airway obstruction symptoms in most cases [12,23], as we have observed in this study.

It is important to note that some pediatric patients may not experience a complete resolution of symptoms after undergoing adenotonsillectomy [14,24]. Our study aligns with previous research and highlights a group of children who still experience airway problems. These residual symptoms may include snoring, mouth breathing, noisy breathing, poor weight gain, poor appetite, and hearing loss.

While preoperative size assessment of tonsils and adenoids remains a valuable tool for understanding the severity of upper airway obstruction, it should not be the sole determinant in predicting postoperative outcomes. Previous studies have investigated various clinical factors associated with persistent symptoms after adenotonsillectomy, such as allergic rhinitis, hypertrophy of the inferior turbinate, regrowth of adenoids, and obesity [16, 20,25]. A more comprehensive evaluation is necessary, considering various clinical factors contributing to symptom persistence after surgery.

It is essential to acknowledge the limitations of the study. One of the limitations was that the data were collected from guardians, which could lead to bias as guardians may report symptoms they consider important or are worried about rather than accurately reflecting the child's symptoms. Another limitation was that the study did not assess the severity of each symptom before and after surgery, meaning that some children may have experienced more significant improvements in symptom severity than others. Lastly, while standardized grading scales were used, the study still relied on subjective airway obstruction assessments, which are generally less reliable than objective assessments.

## Conclusions

This study provides valuable insights into how preoperative tonsil and adenoid grades correlate with the prevalence of airway obstruction symptoms in pediatric patients undergoing adenotonsillectomy. The findings indicate no connection between preoperative adenotonsillar grades and postoperative persistent symptoms. This emphasizes the continued need for research to enhance our understanding of the complexities of adenotonsillectomy outcomes and improve patient care. Furthermore, this study calls for careful, regular postoperative healthcare follow-up for children who experienced symptoms of upper airway obstruction.
